# The role of concentration the young gymnast performance

**DOI:** 10.3389/fspor.2026.1836404

**Published:** 2026-06-30

**Authors:** Helmy Firmansyah, Tri Martini, Mudjihartono

**Affiliations:** Department Physical Education, Health, and Recreation, Faculty of Sports and Health Education, Universitas Pendidikan Indonesia, Bandung, Indonesia

**Keywords:** cognitive performance, concentration, gymnast, gymnastics, performance

## Abstract

**Background:**

In gymnastics, athletes may demonstrate strong technique yet make minor errors from lapses in focus. Others remain attentive but struggle to recall complex movement sequences. This highlights that gymnastics is shaped by both motor and cognitive abilities.

**Objective:**

This research is to investigate the role of cognitive (concentration abilities) in improving the performance of gymnastics athletes.

**Method:**

The research used was correlational, involving seventeen young female artistic gymnasts who consistently participated in training. The research instruments were a concentration grid test and a floor exercise performance test.

**Results:**

Showed that concentration significantly influenced performance, especially in floor exercises.

**Discussion:**

Artistic gymnastics achievement depends not only on physical readiness but also on cognitive readiness. Cognitive readiness supports consistency in movement, technical accuracy, and mental stability in both training and competition. Further research can explore specific cognitive training interventions to better understand the cognitive contribution to gymnastics performance.

## 1 Introduction

Gymnastics demands coordination between body and mind (1). Each movement must follow an exact sequence, with seamless transitions and accurate concentration, to prevent errors. Muscle strength, flexibility, and balance are crucial. Equally important are memory for movement sequences and sustained attention (2). Gymnastics performance depends on both motor skills and cognitive capacity. Cognitive function, or cognitive abilities, improves with physical activity and exercise (3, 4), offering a competitive advantage in sports (5, 26). Performance in sports depends on cognitive abilities. Memory, a key aspect of cognition (6, 7), enables gymnasts to recall movement patterns, maintain smooth transitions, and correct mistakes. Without strong memory, a gymnast is more likely to lose sequence flow, make errors, or fail to finish their routine.

In addition, concentration is another crucial cognitive aspect supporting gymnastics performance. Concentration is the ability to maintain continuous attention on a series of movements while ignoring irrelevant stimuli (8). In gymnastics, concentration determines the accuracy of every movement's execution. These details include body position, rhythm, and the transitions between elements. Athletes who can maintain concentration throughout their routine are more consistent in maintaining high performance quality. In contrast, even minor distractions can lead to mistakes that result in poor performance or increase the risk of injury.

Most studies on gymnastics focus on physical factors, such as strength, flexibility, anthropometry, and coordination, as primary determinants of performance (9–11). The role of cognitive functions such as concentration is less explored. Research linking cognition and athletic performance usually targets sports requiring quick decisions in dynamic settings, such as soccer (12, 13), volleyball (14, 15), handball (16), and basketball (17). Gymnastics, however, is distinct. It requires movement sequences, sustained concentration, and ongoing adaptation to minor errors. Despite research mainly emphasizing physical factors, cognitive functions in gymnastics remain underexplored. This gap suggests a limited understanding of how specific cognitive functions influence gymnastics performance. Thus, this study examines the impact of memory and concentration on gymnastics performance, providing a clearer understanding of the cognitive factors that contribute to athletes' success.

## 2 Material and methods

### 2.1 Participants

This study uses a correlational method, involving 17 selected artistic gymnasts who regularly attend training and will compete in all categories (4 apparatus, team, and all-round event) at the Indonesian National Gymnastics Championships. The sampling technique used was saturated sampling, whereby the entire population of available athletes was used as the research sample. All participants gave their consent prior to data collection. Data was collected when participants were in peak physical condition. Data was collected when participants were in peak physical condition. Detailed physical and performance characteristics are presented (see Table 1).

**Table 1:** Physical and performance characteristics of the subjects.

Data	$\bar{x}$ ± Sd	Min	Max	Level	N
Age	18.70 ± 0.58	17	19	–	17
Height (cm)	1.55 ± 0.56	1.40	1.63	–	
Weight (kg)	51.29 ± 4.67	45	58	–	
Body Mass Index	21.00 ± 2.31	18	25	–	
Duration of Training (year)	12.17 ± 1.09	10	14	–	
Training Duration/Week	20.82 ± 6.54	12	30	–	
Athlete Category	–	–	–	Province/National	

### 2.2 Instruments

This study used several instruments for data collection, including tests of memory, concentration, and gymnastics performance. Memory was assessed using the Digit Span test, which has been validated in various studies (3, 18, 19). The test presents sequences of 3 to 10 digits in forward and backward forms. In the forward test, participants repeat numbers as given; in the backward test, they write them in reverse. Difficulty increases gradually, with two attempts per level. Correct and incorrect answers are marked with a check (√) or X. Concentration was assessed with the Concentration Grid Test (CGT), a 10 × 10 grid of random two-digit numbers from 00 to 99. The test requires a quiet room, a test sheet, a writing instrument, and a stopwatch to measure completion time.

### 2.3 Procedures

Gymnastics performance measurements were conducted by two judges in accordance with the FIG Code of Points. The D Jury was responsible for assessing the degree of difficulty, evaluating elements performed, and determining movement connections. The E Jury focused on rating technical accuracy, assessing artistic presentation, and judging movement quality. The final score was obtained from a combination of the difficulty score assigned by the D Jury and the execution score assigned by the E Jury. Following the gymnastics performance measurements, data analysis was performed using SPSS version 27. Descriptive statistics were presented through means, standard deviations, and minimum and maximum scores. The Shapiro–Wilk normality test showed that the data were normally distributed. Contributions were analyzed using linear regression, with a significance level set at *p* < 0.05.

## 3 Results

**Figure 1:**
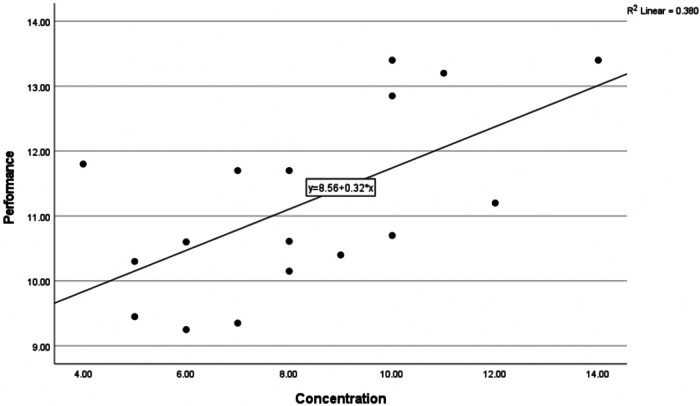
The scatter plot of concentrations’ contribution to gymnastic performance *r*^2^ = .380; *p* = .008.

The research data includes cognitive function scores focused on concentration abilities as well as the performance of gymnasts as research samples (see Table 2). Table 2 shows concentration had an average score of 8.23 with a standard deviation of 2.70, a maximum score of 14, and a minimum score of 4. Meanwhile, gymnastics performance scores had an average score of 11.18 with a standard deviation of 1.39, a maximum score of 13.4, and a minimum score of 9.25. The contribution of concentration to gymnastics performance (see Table 3). The coefficient of determination value (*r*^2^ = .502; *p* = .001) indicates that concentration contributes 50.2% to gymnastics performance. This result is reinforced by the scatter plot visualization, which displays a positive linear pattern in the data distribution. This means that the higher the concentration abilities, the better the gymnastics performance achieved by the athlete. Concentration skills significantly impact gymnastics performance. The analysis reveals that concentration accounts for approximately 38.0% of the performance variation (*r*^2^ = 0.380; *p* = 0.008). A scatter plot (see Figure 1) illustrates this positive relationship, indicating that higher concentration levels are associated with improved movement quality and accuracy. The results show a coefficient of determination (*r*^2^ = 0.515; *p* = 0.006), indicating that these cognitive aspects (concentration) explain 51.5% of the variation in performance. Concentration is very important individually in improving athlete performance and help remember a sequence of movements and stay focused during execution. This combination leads to better results.

**Table 2:** Descriptive data concentration and performance of gymnast athletes.

Variable	$\bar{x}$ ± Sd	Min	Max
Concentration	8.23 ± 2.70	4	14
Performance	11.18 ± 1.39	9.25	13.4

**Table 3:** The linear regression test on concentration and gymnastic performance.

Variable	Contribution			*p*
	r	r^2^	%	
C	.708	.502	50.2	.001
P	.617	.380	38	.008
C&P	.717	.515	51.5	.006

C, concentration; P, performance; C&P, concentration and performance.

## 4 Discussion

A gymnastics performance exemplifies the intricate interaction of physical skills and cognitive processes. Beyond physical elements such as strength, flexibility, and balance, gymnasts navigate substantial cognitive challenges during routines. They memorize predetermined movement sequences and sustain focused attention throughout, underscoring the critical influence of cognitive functions on performance quality (20, 21). Concentration acts as the gateway to memory. It directs your attention to process new information, allowing your brain to encode it from sensory memory into working memory. Without sustained focus, the brain cannot form lasting neural connections, making concentration the critical first step for effective learning and long-term retention. Memory and concentration are functions carried out by our brain in learning processes and assimilation. When learning something new, new neural connections can be established, facilitating memories both in the short term and long term. Both functions are complementary and necessary throughout our lives, but especially at specific times such as during the period of training and study or demanding work periods, and even adapting to new environments.

In motor learning, memory—especially procedural and working memory—stores and retrieves movement representations needed for skilled performance (6, 7). Previous research has shown that memory significantly affects motor control and skill automation, particularly in sports that involve repetitive, complex movements (22, 23). In gymnastics, concentration is a very important cognitive component. Concentration refers to the ability to maintain attention on cues relevant to the task while regulating internal and external distractions during performance (8). In the context of gymnastics, sustained concentration allows athletes to maintain body alignment, timing, and movement control throughout all phases of the routine, both during training and competition. The importance of concentration in maintaining consistent performance is widely acknowledged in sports psychology literature, particularly in precision-based sports that demand sustained attention (24, 25). Even brief lapses in concentration can lead to technical mistakes that compromise performance quality. Thus, concentration acts as a regulatory mechanism, ensuring accurate execution and fluid movement in gymnastics.

Overall, concentration functions together to support performance, maintaining execution and attention. In gymnastics, where structured, sequential, and precise routines are vital, this integration is crucial. Thus, performance differences reflect each gymnast's ability to manage physical and cognitive demands.

## 5 Conclusions

This study demonstrates that cognitive functions, specifically concentration, are significantly correlated with gymnastics performance. The sequential and precision-based characteristics of gymnastics movements require athletes to remember movement patterns and maintain attention control during execution. These findings support the idea that performance in gymnastics is shaped not only by physical and technical capacity but also by the cognitive readiness of athletes during movement execution.

The results of the study highlight concentration as a crucial factor in supporting the organization and continuity of movement sequences, while concentration contributes to the stability and accuracy of movement execution throughout. The interaction between these cognitive components demonstrates that effective performance stems from the integration of motor and cognitive processes, underscoring a multidimensional perspective on gymnastics performance.

However, this study has limitations. Its correlational design prevents causal conclusions about how cognitive function (concentration abilities) affects performance. Additionally, the specific sample may limit the generalizability of these findings to other age groups, competitive levels, or gymnastics disciplines. Practically, these findings suggest coaches and practitioners should consider cognitive factors, such as concentration, in athlete development and performance evaluation. Further research should investigate longitudinal or intervention-based approaches to elucidate the relationship between six domain cognitive function (complex attention, executive function, learning and memory, language, perceptual-motor control, and social cognition) and technical training, and how it enhances gymnastics performance in all apparatus, with a larger sample size.

## Data Availability

The raw data supporting the conclusions of this article will be made available by the authors, without undue reservation.
